# Innate immune responses in RNA viral infection

**DOI:** 10.1007/s11684-020-0776-7

**Published:** 2020-12-01

**Authors:** Qian Xu, Yuting Tang, Gang Huang

**Affiliations:** 1 Divisions of Pathology and Experimental Hematology and Cancer Biology, Cincinnati Children’s Hospital Medical Center, Cincinnati, OH 45229 USA; 2 Department of Hematology, Tongji Hospital, Tongji Medical College, Huazhong University of Science and Technology, Wuhan, 430030 China

**Keywords:** innate immune, viral infection, intercellular signaling, metabolic changes, epigenetic changes

## Abstract

RNA viruses cause a multitude of human diseases, including several pandemic events in the past century. Upon viral invasion, the innate immune system responds rapidly and plays a key role in activating the adaptive immune system. In the innate immune system, the interactions between pathogen-associated molecular patterns and host pattern recognition receptors activate multiple signaling pathways in immune cells and induce the production of pro-inflammatory cytokines and interferons to elicit antiviral responses. Macrophages, dendritic cells, and natural killer cells are the principal innate immune components that exert antiviral activities. In this review, the current understanding of innate immunity contributing to the restriction of RNA viral infections was briefly summarized. Besides the main role of immune cells in combating viral infection, the intercellular transfer of pathogen and host-derived materials and their epigenetic and metabolic interactions associated with innate immunity was discussed. This knowledge provides an enhanced understanding of the innate immune response to RNA viral infections in general and aids in the preparation for the existing and next emerging viral infections.

## Introduction

An RNA virus is a virus that uses RNA as its genetic material; it could be single-stranded RNA (ssRNA) or double-stranded RNA (dsRNA) [1]. Retroviruses are not generally considered RNA viruses because they use DNA intermediates to replicate. However, they have a single-stranded RNA genome. Notable human diseases caused by RNA viruses include coronavirus disease 2019, severe acute respiratory syndrome (SARS), Ebola virus disease, common cold, influenza, hepatitis C, hepatitis E, rabies, polio, and measles. RNA viruses generally have higher mutation rates than DNA viruses due to the lack of proofreading ability of the RNA polymerases in contrast to DNA polymerases, hindering the development of optimal vaccines to prevent the diseases caused by RNA viruses.

Upon viral infection, the innate immune system acts as the first line to prevent the spread of the invading pathogens and plays a crucial role in triggering the adaptive immunity. The innate immune cells could recognize the conserved features discriminately expressed by the virus but not on the host cells as pathogen-associated molecular patterns (PAMPs). Through interactions with PAMPs, the pattern recognition receptors (PRRs) expressed by innate immune cells activate several intracellular signaling pathways and induce the production of type I interferons (IFNs) and pro-inflammatory cytokines to initiate the antiviral responses of the host [2,3]. In a prototypical response to an acute viral infection, the clearance of viral-infected cells is expected to be achieved within a few weeks. However, some viruses develop strategies to inhibit or evade the host immune responses that favor their replication and cause persistent infection in the host. The molecular mechanisms, under which the viruses evade the antiviral immune responses and establish their persistence in the host, remain to be further investigated.

The interactions between the invading virus and the innate immunity are complex, and they consist of multiple layers. This review highlighted the recognition and defense mechanisms adopted by innate immune cells against RNA viral infection, with a focus on cell type-specific cellular responses. The intercellular transfer of pathogen and host-derived material, which is a newly discovered mechanism affecting the innate immune signaling, was also discussed. Moreover, the epigenetic interactions between the host and the RNA viruses were briefly introduced. These interactions are also new to the field, and they broaden the understanding of antiviral responses in innate immunity. Finally, the cellular metabolic alterations affected by the IFN-generated antiviral state, as indicated by recent evidence and previous observations, were explored.

## Viral recognition and initiation of antiviral responses

Upon the invasion of viruses, the innate immune system is the first line of host defense and is rapidly activated. The innate immune cells sense the viral invasion through multiple types of ligand–receptor interaction [4]. PAMPs are conserved structural motifs that could activate innate immunity to protect against infections through recognition of properties derived from viruses or bacteria that distinguish the pathogen components from the host, including nucleic acids, peptides, and lipoproteins. During viral infection, PAMPs could be recognized by host PRRs expressed not only in innate immune cells, such as macrophages, dendritic cells (DCs), and natural killer (NK) cells but also in other cell types, such as epithelial cells. PRRs consist of the membrane-bound type and the cytoplasmic type. The former includes Toll-like receptors (TLRs) and C-type lectin receptors (CLRs), while the latter includes retinoic acid-inducible gene 1 (RIG-I)-like receptors (RLRs), nucleotide oligomerization domain-like receptors (NLRs), and cyclic GMP-AMP synthase (cGAS). This section focused on the PRRs that mediate the signaling responses in innate immune cells during RNA viral infection.

TLRs are a class of transmembrane receptors expressed by antigen-presenting cells (APCs), such as DCs and macrophages, and some types of T cells (Table 1). Unlike the TLRs recognizing bacterial components mainly expressed on plasma membrane, the human TLRs, such as TLR3, TLR7, TLR8, and TLR9, that could recognize viral nucleic acids are expressed exclusively in the endosomal compartment. Double-stranded viral RNA taken up into the endosomes of sentinel cells are sensed by TLR3 [5–8] to recruit the cytoplasmic adaptor protein TIR domain-containing adaptor inducing IFN-β (TRIF)-dependent downstream signaling pathways. ssRNAs are sensed by TLR7 and TLR8, which utilize MyD88-dependent downstream signaling pathways [9]. TLR9 is the only known DNA sensor that recognizes the unmethylated CpG DNA of DNA viruses [10]. However, TLR9-mediated signaling may also play a role during RNA viral infection, as revealed by the recent finding that dengue virus (DENV) infection could induce a release of mitochondrial DNA into the cytosol and activate TLR9 signaling pathways [11].

RLRs are a group of cytosolic RNA helicases that detect RNA species in the cytoplasm of infected cells [12]. To date, three receptors have been identified: RIG-I, melanoma differentiation-associated gene 5 (MDA5), and laboratory of genetics and physiology 2 (LGP2). These RLR proteins are distinct in their RNA recognition capacities and signaling properties. Functional domains shared by these proteins are the DExD/H box RNA helicase domain, which could bind to dsRNA and induce ATP hydrolysis, and the C-terminal domain (CTD), which is associated with binding to RNA termini and autoregulation [13]. Only two N-terminal tandem caspase recruitment domains (CARDs) are presented by RIG-I and MDA5 but not by LGP2, and they are crucial for the activation of downstream signaling. MDA5 senses long dsRNA without unpaired bulged nucleotides, while RIG-I senses short 5′ tri- and dephosphorylated dsRNA with base-paired terminal 5′ and 3′ nucleotides [14–16]. Then, oligomerization of mitochondrial antiviral signaling protein (MAVS) is triggered [17,18]. LGP2 functions as regulators for the other two receptors, inhibiting RIG-I-mediated responses while enhancing MDA5 signaling [19]. This differential effect was recently found to be dependent on a direct protein–protein interaction between the regulatory CTD of LGP2 and the IFN-inducible, double-stranded RNA binding protein PACT [19]. LGP2 is at low levels in uninfected cells but accumulates after the viral infection or due to the treatment of poly(I:C) and IFN [20,21].

CLRs could recognize various carbohydrate ligands with their carbohydrate recognition domains. In addition to the initiation of inflammatory mediator production, pathogen sensing by CLRs may initiate phagocytosis or innate killing depending on the specific CLR and cell type [22]. The mannose receptor (MR) is one type of CLR expressed on macrophages and DCs to recognize mannose, fucose, and N-acetylglucosamine on the surfaces of viruses, bacteria, and parasites [23]. Through its activation, the pathogen could be internalized and targeted to lysosomes for degradation. However, evidence also suggested that DENV utilizes MR to evade the degradation after internalization [24]. Thus, the role of CLRs could either be routing for the degradation or the dissemination of viruses.

The signaling pathways downstream of PRRs lead to the expression and/or activation of transcription factors, such as NF-κB, AP-1, and interferon regulatory factors (IRFs), which all induce the expression of downstream anti-viral genes, including types I and III IFNs, pro-inflammatory cytokines, and chemokines. These target genes could enhance the adaptive immune response, inhibit pathogen replication, and adapt the host cells to environmental changes [25–29]. Type I IFNs, which are mainly produced by macrophages and DCs [30–32], induce an anti-viral state in the neighboring cells by stimulating the expression of hundreds of genes that are collectively known as IFN-stimulated genes (ISGs). The chemokines produced at the location of infection could recruit additional immune cells, including neutrophils, monocytes, and NK cells (Fig. 1).

NLRs could detect a wide range of PAMPs and DAMPs. NLR family pyrin domain containing 3 (NLRP3) is an important component of the inflammasome system and the most well-studied NLR. It could be detected in myeloid cell types, including monocytes, macrophages, DCs, and neutrophils [33]. Many pathogens, including DNA and RNA viruses, fungi, and protozoa, could activate the NLRP3 inflammasome and induce the maturation of inflammatory cytokines IL-1β and IL-18 [34–36], resulting in inflammatory pyroptotic cell death and the formation of phagosomes. Some viruses even induce abnormal activation of the inflammasome and aggravate the diseases. SARS coronavirus (SARS-CoV) is an enveloped positive-strand RNA virus that encodes a number of accessory proteins, including open reading frame (ORF) 8a, 8b, and 9b. SARS-CoV triggers robust NLRP3 inflammasome activation and IL-1β release by direct binding of ORF8b to the LRR domain of NLRP3. The aberrant inflammasome activation and cytokine storm lead to excessive inflammation and enhanced disease [37]. ORF8b and 8ab are also confirmed as novel IFN antagonists that limit IRF3 activation and/or promote proteasome-mediated degradation of IRF3 [38].

A major breakthrough in 2013 discovered cGAS could bind directly to viral DNA, trigger conformational rearrangement, and catalyze cyclic GMP-AMP (cGAMP) synthesis, which activates stimulator of IFN gene (STING)-dependent TBK1-mediated IRF3 axis or IKKs-mediated NF-κB axis [39,40]. DNA viruses are targeted by this pathway, while some RNA viruses could manipulate the DNA sensors, such as cGAS/STING, in turn. For example, Zika virus-encoded NS1 protein is able to recruit caspase-1 activation and induce proteolysis of cGAS [41]. This interplay between inflammasome and cGAS/STING pathways facilitates the immune escaping strategy of Zika virus.

## Important components shaping innate immune responses: macrophages, DCs, and NK cells

Within minutes to hours upon the detection of pathogens, tissue-resident macrophages and DCs are activated. Then,

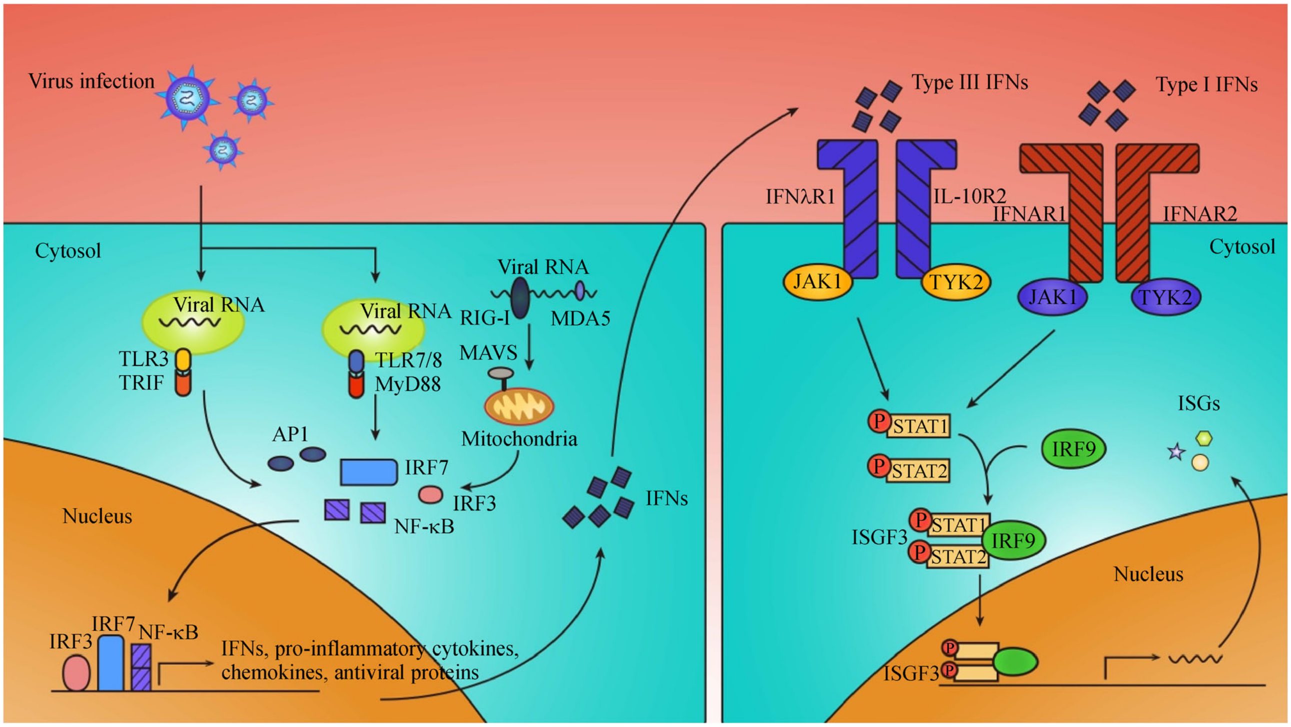

**Fig. 1** Schematic of RNA viral signaling pathway. Endosomes expose viral RNAs to TLR3, 7, and 8. The TLR signaling pathways consist of two major cascades, namely, MyD88 and TRIF, depending on TLR domain-containing adaptors. RIG-I and MDA5 could sense RNA in the cytoplasm and trigger the oligomerization of MAVS. These transduction cascades result in IRF3/7 and NF-κB activation, which induces IFN secretion. Types I and III IFNs are sensed by distinct membrane-bound receptor complexes and stimulate similar signaling pathways. IFNs could activate the transcription of ISGs through intracellular kinases JAK1 and TYK2 and induce signaling pathways through STAT1/STAT2/IRF9 binding to the IFN-stimulated response elements (ISREs) in target gene promoters. Subsequently, a large number of ISGs is activated.

**Table 1** PRRs in innate immune cells and PAMPs

| PRRs | | PAMPs | References |
|---|---|---|---|
| TLRs (transmembrane) | TLR1 | Lipoproteins (bacteria) | [42,43] |
| | TLR2, TLR4 | Structural proteins (capsid, envelope proteins) | [42–45] |
| | TLR3 | dsRNA | [5–8] |
| | TLR5 | Flagellin (bacteria) | [46] |
| | TLR6 | Lipoproteins (bacteria) | [42] |
| | TLR7 | ssRNA | [9] |
| | TLR8 | ssRNA | [9] |
| | TLR9 | CpG motif (bacteria, virus) | [10] |
| | TLR10 | Not determined | |
| RLRs (cytoplasm) | RIG-I | RNA | [17,18] |
| | MDA5 | RNA | [17,18] |
| | LGP2 | RNA | [20,21] |
| NLRs (cytoplasm) | NLRP3 | DNA, RNA, protein | [33] |
| | NLRP1 | ssRNA | [47] |
| | NLRP9b | dsRNA | [47] |
| CLRs (membrane bound) | MMR | Mannose, fucose | [23] |
| Others | STING | DNA | [39,40] |

the inflammatory cytokines, chemokines, biogenic amines, and eicosanoids produced by these two types of cells recruit additional innate immune cells, including NK cells, neutrophils, and monocytes, to the infection locations.

## Macrophages

Macrophages evolve in the innate immune system and play important roles in the regulation of inflammation and phagocytosis of viruses [48–50]. However, they are also well-known for their "double-edged sword" role in the immune system due to their versatile polarization states, which depend on the converging signals from inflammatory stimuli in the environment. The versatile states exert multiplex functions, including pathogen elimination, pro-inflammation, and tissue destruction and repair [51,52]. M1 type macrophages, such as M(IFN-$\gamma$), M(LPS + IFN-$\gamma$), and M(LPS) subtypes, present as proinflammatory, tissue-destructive, antitumoral, antimicrobial, and immunogenic macrophages. By contrast, M2 type macrophages such as M(IL-4), M(Ic), M(IL-10), M(GC + TGF-$\beta$), and M(GC) subtypes, are associated with viral persistence and could promote tissue repair in some cases [49,50,52–54].

Macrophages express receptors for all three types of IFNs, which are produced by several innate immune cells during viral infection to induce gene expression in the infected and neighboring uninfected cells. Types I and III IFNs are sensed by the receptors of IFNAR1/IFNAR2 and IFNR$\lambda$1/IL-10R2, respectively, leading to the activation and dimerization of STAT1 and STAT2 [55]. Afterward, IRF9 is recruited to form an IFN-stimulated gene factor-3 complex. This canonical signaling pathway induces ISGs and pro-inflammatory responses featuring an M1 status. In addition, type I IFNs could signal through STAT3 and STAT6 homodimers to induce an M2 status [56,57]. Non-canonical signaling pathways, including mitogen-activated protein kinase cascade and PI3K/Akt/mTOR signaling, could be regulated by either type I or type III IFNs [58–63].

Besides IFNs, some RNA viruses themselves affect the macrophage polarization status and compromise their functions. Some coronaviruses could over-activate macrophages to incite M1-associated inflammation, which causes macrophage depletion via apoptosis and necrosis [64–66]. Meanwhile, the incited acute cytokine storm may also harm the host [67]. Targeting the signaling pathways to moderate the virus-induced cytokine storm protects patients from infection, even without suppressing viral replication [68]. RNA viruses, such as hepatitis C virus (HCV), could upregulate IL-10 expression, which induces M2 polarization of macrophages and shows immunosuppressive effects [69–71]. Therefore, these viruses could suppress the anti-viral responses of the hosts and develop persistent and systemic infections [72,73]. In sum, the future therapeutic strategy against viral diseases could be expanded to regulating the polarization status of macrophages rather than solely focusing on killing the viruses [74–76].

## DCs

As unique immune sentinels, DCs play a crucial role in sensing pathogens and inducing immune responses. They are the major APCs in the immune system, and they have different subtypes on the basis of their location, phenotype, and function [77,78]. Human DCs can be divided into two groups: myeloid DCs (mDCs) and plasmacytoid DCs (pDCs) [79]. mDCs mainly reside in lymphoid tissues, such as in spleen, thymus, and secondary lymph nodes. They express myeloid antigens (CD11c, CD13, CD33, and CD11b) and can be further classified into CD1c$^+$ and CD141$^+$ DCs. In tissues and lymph nodes, a third subgroup of mDCs has been detected, and it is defined as interstitial DCs on the basis of CD14 expression. Interstitial DCs were first identified in the interstitium of non-lymphoid organs, and they are closely related to lymphoid DCs. pDCs are a small subset of DCs entering the lymph nodes from the circulatory blood. They do not have myeloid antigens expressed on their membrane. Instead, pDCs express CD123, CD303, and CD304 [79,80].

When exposed to viruses, human DCs sense the molecular patterns through specific receptors, including TLR7 and TLR9, and become mature with the upregulation of CCR7 and MHC molecules. Then, these mature DCs migrate to local-draining lymph nodes and interact with naïve T cells in the secondary lymphoid organs to elicit the adaptive immune response [81].

pDCs play a key role in the early anti-viral responses, primarily because they could produce types I (IFN-α and IFN-b) and III (IFN-λ/IL-28/IL-29) IFNs. In addition, activated pDCs produce proinflammatory cytokines, such as tumor necrosis factor α (TNF-α), IL-6, IL-8, immunomodulatory cytokines, and chemokines. pDCs could also promote the maturation of APCs and activate T cells and NK cells [82]. For some viruses, the resolution of infection is highly correlated with the functions of pDCs. In patients infected with DENV, viral load and disease severity are inversely associated with the number of circulating pDCs and their IFN responses [83].

The role of DC-NK interaction is important in inducing the adaptive immune response to viral infections. MHC class I-related chains A and B (MIC-A/B) expressed on DCs could activate NK cells by transducing positive signals to NK cells. However, chronic HCV-infected DCs, for instance, have decreased MICA/B expression on DCs and production of IL-15. Therefore, patients with chronic HCV infection also have low NK cells [84,85].

## NK cells

NK cells were originally identified by their ability to lyse tumor cells *in vitro* [86]. The antiviral activities of NK cells consist of the production of proinflammatory cytokines, such as IFN-γ, and directing lysis of infected cells [87]. NK cells act as a crucial and early source of IFN-γ, which helps the host defend against viruses by improving the antiviral T cell responses and increasing the non-cytolytic control of viral replication [88,89]. In addition, other cytokines (e.g., TNF-α), inflammatory chemokines (e.g., RANTES), and growth factors could be generated by NK cells [90,91]. Through secreting chemokines and cytokines, NK cells communicate with their neighboring cells in several immunological processes, including viral defense and immunological homeostasis. The direct lysis of infected cells by NK cells is mediated by antibody-dependent cellular cytotoxicity (ADCC). Most NK cells express immunoglobulin G receptor Fcγ RIII (CD16), which mediates the interaction with antibody-coated target cells and activates ADCC.

During viral infection, NK cells recognize inflammatory signals using several strategies. The cytokine–cytokine receptor axis plays an important role in the activation of NK cells. The expression of cytokine receptors for IFN-α, IL-12, IL-15, and IL-18 are upregulated early after the infection [92]. The above cytokines could provide stimuli to imprint the stable expression of IFN-γ in NK cells. The IFN-γ expression in NK cells represents their expansion capacity [93,94] and increased cytotoxicity [95]. Moreover, the activation of cytokine receptors protects NK cells from "fratricidal" killing via NK cell population expansion and defense against viruses [96].

The balance between inhibitory and activating receptors expressed on NK cells is modulated during viral infection. The major inhibitory receptors include killer cell immunoglobulin-like receptors (KIRs) and CD94/NKG2A. The binding of these inhibitory receptors to MHC class I molecules maintains the tolerance of NK cells in healthy host cells [97]. During tumor development or viral infection, NK cells detect modified host cells through a "missing self" mechanism (MHC class I protein downregulation), which represents a central regulatory pathway during NK cell activation [98]. NK cells also have activating receptors (e.g., NKG2D, NKG2C, and DNAM-1) expressed on their membrane [97,99–101] that could initiate rapid killing of target cells [102,103]. NKG2D could be upregulated by various viral infections and provide an activating signal upon recognition of stress-induced ligands on infected cells [104]. This "induced-self" recognition helps NK cells to clear harmful host cells. However, some viruses could evade the host's immune system by preventing the upregulation of NKG2D ligands expressed on the infected cells [105,106]. NKp46 activates NK cells in response to viral ligands encoded by parainfluenza [107], influenza A virus [108], metapneumovirus [109], and reovirus [110]. NKG2C is only expressed on a specific population of NK cells, which recognize pathogen-derived antigens. Compared with conventional NK cells, NKG2C-positive NK cells expand in patients with HCV infection or dengue viral infection only when the patients are HCMV-seropositive and display different transcriptomic signatures (Fig. 2) [111–114].

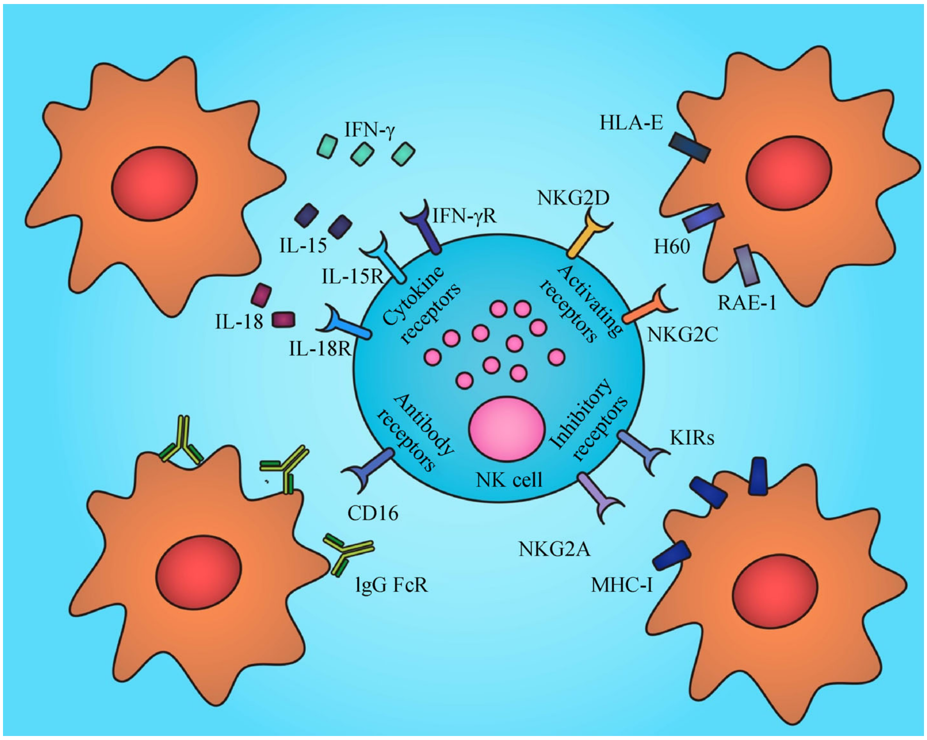

**Fig. 2** NK cell surface receptors. NK cell surface receptors are broadly classified into four types: cytokine receptors, antibody receptors, activating receptors, and inhibitory receptors. When viral infection occurs, NK cells could be activated by cytokines, such as type I IFNs or IL-18 secreted by accessory cells. They could also be activated by antibody-coated cells that crosslink immunoglobulin G receptor CD16. KIRs and CD94/NKG2A are the major inhibitory receptors on NK cells. In addition, NK cells have activating receptors (e.g., NKG2D and NKG2C) expressed on their membrane that could initiate rapid killing of target cells.

## Intercellular innate immune signaling

Intercellular communication and secretion of cytokines/chemokines ensure fast and efficient responses to the threats from the environment surrounding the host cells. Besides the classical innate immune signaling, the transfer of PAMPs and host-derived signaling molecules from the infected host cells to the neighboring non-infected cells serves as an important alternative pathway. This process is an essential component in viral uptake, especially during the inhibition of innate immunity induced by persistent infection. It also helps the virus evade the host immune system through immunoregulatory mechanisms [115,116].

Intercellular communication mainly occurs through the cellular release of extracellular vesicles (EVs). Exosomes are a type of EVs ranging from 30 nm to 100 nm in diameter; they contain host (or pathogen)-derived nucleic acid, protein, and lipid cargos. These contents could be captured in the cytosol via endosomal membrane invagination and enriched by interacting with endosomal sorting complexes required for transport (ESCRTs) [117]. In the context of viral infections, exosomes containing viral mRNAs and microRNAs (miRNAs) could be released into extracellular spaces and detected in biological fluids, such as blood, cerebrospinal fluid, urine, semen, and breast milk. These exosomes mediate immune responses by regulating the production of type I IFNs in the surrounding uninfected cells and control the viral dissemination [118–120].

HCV is an ssRNA virus. HCV-infected cells induce the production of type I IFNs of uninfected pDCs through a cell-to-cell RNA transfer mechanism rather than virus–particle assembly and virus–particle release [121]. A follow-up study showed that this process was mediated by the release of exosomes, which could be suppressed by exosome release inhibitors [122]. Therefore, exosomal RNA transfer is an important mechanism for the activation of innate immunity during infection.

Besides the PAMP exportation during infection, host PRR proteins can also be transferred via cell-to-cell interaction. The inflammasome is a cytoplasmic protein complex responding to pathogens and warning signals. It contains the innate immune sensor and the adaptor molecule apoptosis-associated speck-like protein containing a CARD (ASC), which generates a “speck” in the cytoplasm. Caspase-1 is then recruited to cleave and activate IL-1β and IL-18 [123,124]. The inflammasome complex can remain stable and last for up to 72 h *in vitro*

[125]. If there is proIL-1β and proIL-18 available extracellularly, they will activate the inflammasome complex and induce inflammation [126,127]. Moreover, the infected and dying cells can transfer these signals, such as inactive substrates, to neighboring cells. It indicates that the pathogen is detected through the extracellular ASC specks phagocytosis followed by activation of an additional anti-microbial immune response by neighboring cells. However, some pathogens also destroy these mechanisms to benefit their survival and prolong their presence in host cells [118,128] (Fig. 3). It is still unclear how far these intercellular communications can spread and how to control it. Nonetheless, this type of communication in the innate immune system may also provide potential therapeutic strategies for viral infectious diseases.

## Metabolic changes in innate immunity after viral invasion

Viruses generally do not have their own metabolism as they are not living entities. They could induce alterations in cellular metabolism, such as increasing glycoproteins for the envelope of the virus, nucleotides for viral genome replication, and amino acid for virion assembly, to gain specific materials required for virion production. In addition, the altered cellular metabolism may improve the survival of infected cells aiding the persistence of the virus [129–133]. The altered core cellular metabolic pathways include glycolysis, fatty acid synthesis, and glutaminolysis. HCV infection in Huh7 cells increases glucose requirement and decreases host cell oxidative phosphorylation [134]. Increased expression of many glycolytic enzymes in Huh7 cells with HCV infection was shown in a global proteomic screen [131]. In addition, transcriptomic studies suggested that HCV microRNA miR-146a-5p could upregulate the transcription of genes related to fatty acid metabolism [135].

These alterations in host cells are mainly driven by IFNs to form an antiviral state and elicit subsequent immune responses. IFNs could affect the amino acid metabolism of cells by depleting polyamines and stimulating arginine-dependent nitric oxide (NO) production. Polyamine, which is generated from amino acid ornithine decarboxylation, consists of three molecules: putrescine, spermidine, and spermine [136]. It is associated with the process of deacetylation, transcription, and translation and affects cell proliferation, autophagy, and apoptosis. Putrescine is

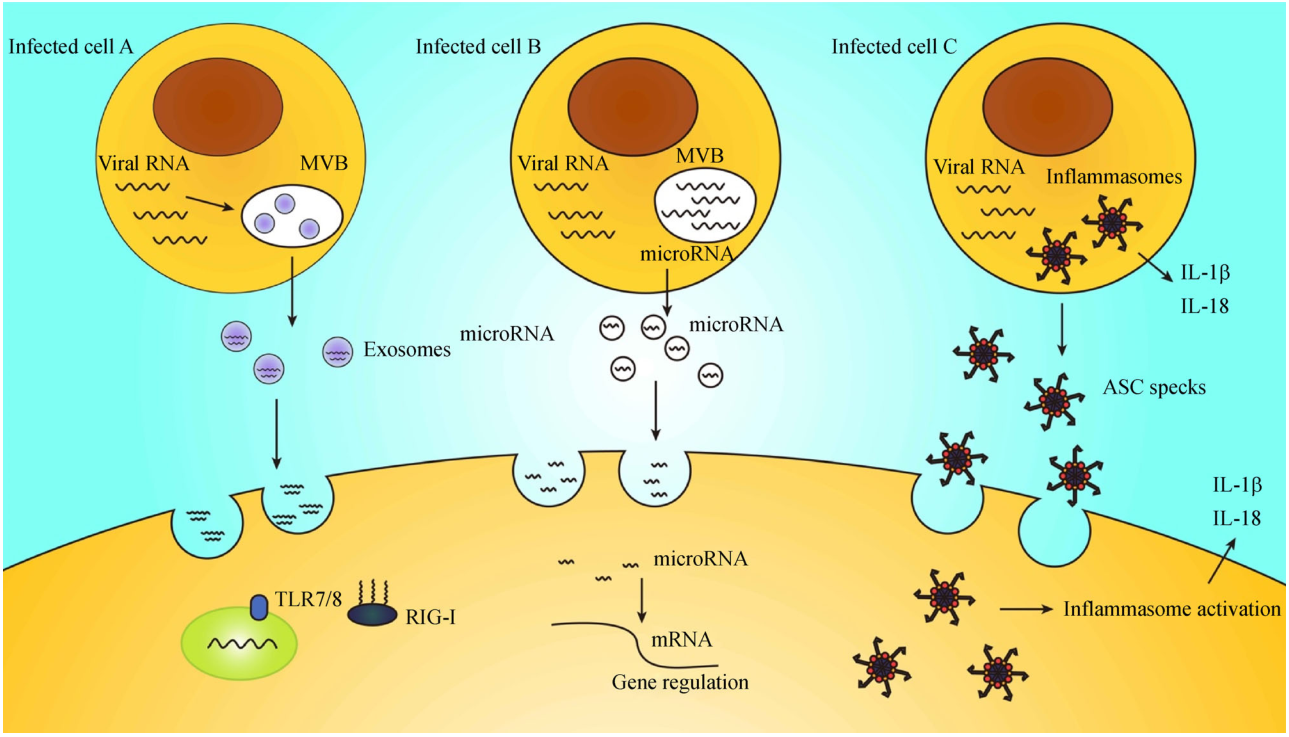

**Fig. 3** Intercellular transfer of virus-derived RNAs, microRNAs (miRNAs), and inflammasome complex leading to bystander activation of innate immunity. Virus-derived RNAs, including miRNAs, are produced in the infected cells and packaged into exosomes, which are released into the extracellular space. Then, the RNAs are internalized by the uninfected cells via endocytosis and activate TLRs and RIG-I to induce the production of type I IFN. Inflammasome activation could induce IL-1β and IL-18 production and pyroptosis, which is a type of cell death that causes inflammasome complex release and phagocytosis by neighboring cells to induce inflammasome activation.

generated from ornithine, which is processed from L-arginine by arginase [137]. Putrescine could also generate spermine. Type I IFN induces the spermidine-spermine acetyltransferase (SAT1) expression and decreases the expression levels of spermidine and spermine by acetylating them [136,138]. This reduction could inhibit RNA viral replication, as polyamines are essential for the transcription and translation of viral RNA and protein.

Moreover, IFNs stimulate the production of NO, which mediates the inhibition of viral replication and leads to enhanced clearance of the pathogen [139]. NO synthase (NOS) type 2 (iNOS) is induced by IFN-$\gamma$ and regulates the switch from L-arginine to L-citrulline to produce NO [139,140]. One of the antiviral activities of NO is the nitrosylation of viral molecules [141]. For instance, NO inhibits the activity of Coxsackievirus protease 3C by nitrosylating cysteine residue and disturbing the viral life cycle [142]. In addition, the NO generated by iNOS could deplete L-arginine and then reduce polyamines. Therefore, iNOS induced by IFN has antiviral effects through a shift from polyamine synthesis to NO production [143].

However, types I and II IFNs could also result in tissue damage due to the overproduction of reactive oxygen species and an increase in the apoptosis of overactivated immune cells [144–146]. In addition, large amounts of IFN-activated macrophages generate large amounts of NO, which also contributes to tissue damage [147]. In summary, the IFN-induced metabolic effects constitute one of the central antiviral mechanisms and suggest potential novel metabolic therapies for viral infectious diseases.

## Epigenetic changes in innate immunity after viral invasion

Some viruses have developed several epigenetic strategies to replicate and survive in hosts, including taking advantage of pathogen-specific gene products to modify host proteins and chromatin, repressing PRR sensing and signaling pathways, and adjusting the expression of activators and repressors in innate immunity. Hosts also antagonize pathogen-induced changes in epigenomes to maintain an effective antipathogen immunity. Epigenetic regulation consists of DNA modifications, histone post-translational modifications, chromatin remodeling, and non-coding RNAs. These epigenetic regulations involve the host and the virus.

Besides the epigenetic changes in innate immune signaling pathways and effector molecules driven by these main epigenetic regulators, pathogen-mediated epigenetic dysregulation to the host chromatin could help the virus with immune evasion, infection persistence, and inflammation [148]. Viruses mediating host chromatin modifier changes or encoding epigenetic regulators could cause the dysregulation of host chromatin in the infection. Respiratory syncytial virus (RSV) infection induces the upregulation of H3K4 demethylase KDM5B and represses type I interferons and the production of other innate cytokines [149]. The RNA viruses of the Flaviviridae family could change the m6A levels in host mRNAs [150]. These viruses use different mechanisms to alter the host's epigenome by targeting regulators for DNA and RNA methylations. Viruses could modify chromatin at a specific host gene locus. SETDB2, an H3K9me3 methyltransferase that triggers H3K9me3 at gene promoters, could be induced by the influenza virus and inhibit neutrophil attractant CXC-chemokine ligand 1 and a subset of NF-κB-inducible genes [151]. Moreover, the epigenetic dysregulation in immune-related diseases promotes viral infection. Airway diseases, such as asthma, upregulates TGFβ expression, which mediates the epigenetic reprogramming of lung epithelium known as epithelial-to-mesenchymal transition. The transition induces the ZEB1 silencing of IRF1 expression, which leads to type III interferon silencing and enhanced RSV infections [152].

Chronic infection of some oncogenic viruses also interacts with epigenetic dysregulation to persist in the host and intensify infectious diseases and cancer pathogenesis. For instance, the microbial signals of chronic inflammation could induce pre-leukemic myeloproliferation in a host with TET2 mutations by inhibiting cytokine expression [153,154].

These chromatin modifiers affect the host and pathogen genes, which in turn affects the host's effective clearance and the pathogen's immune evasion. Thus, targeting these epigenetic modifiers represents potential novel therapies for viral infectious diseases.

## Conclusions and perspectives

This review briefly discussed the role of the innate immune response in combating RNA viral infection and how several aspects of the innate cellular responses contribute to host protection. Different immune cells in the innate immune system have unique characteristics, and they utilize various mechanisms to eliminate viruses. This review did not focus on each aspect in depth but rather highlighted their commonality, differences, and interdependence. Clarifying the underlying mechanisms of how viruses evade innate immunity is considerably important for the understanding of viral pathogenesis and the preparation for the next emerging viral disease. Knowledge regarding mediators, such as signaling, metabolism, and epigenetics of the innate immune system, adds to the understanding of their contribution to innate immune responses and may lead to novel targets for the treatment of human viral infectious diseases.

## Compliance with ethics guidelines

Qian Xu, Yuting Tang, and Gang Huang declare no conflict of interest. This manuscript is a review article. It does not involve a research protocol requiring approval by relevant institutional review board or ethics committee.

**Open Access** This article is licensed under a Creative Commons Attribution 4.0 International License, which permits use, sharing, adaptation, distribution and reproduction in any medium or format, as long as you give appropriate credit to the original author(s) and the source, provide a link to the Creative Commons license, and indicate if changes were made.

The images or other third party material in this article are included in the article's Creative Commons license, unless indicated otherwise in a credit line to the material. If material is not included in the article's Creative Commons license and your intended use is not permitted by statutory regulation or exceeds the permitted use, you will need to obtain permission directly from the copyright holder.

To view a copy of this license, visit https://creativecommons.org/licenses/by/4.0/.
